# Genetic diversity estimates for the *Caenorhabditis* Intervention Testing Program screening panel

**DOI:** 10.17912/micropub.biology.000518

**Published:** 2022-01-27

**Authors:** Anastasia A Teterina, Anna L Coleman-Hulbert, Stephen A Banse, John H Willis, Viviana I Perez, Gordon J Lithgow, Monica Driscoll, Patrick C Phillips

**Affiliations:** 1 Institute of Ecology and Evolution, University of Oregon, Eugene, OR, 97403, USA; 2 Center of Parasitology, Severtsov Institute of Ecology and Evolution RAS, Moscow, Russia; 3 Division of Aging Biology, National Institute on Aging, Bethesda, MD, 20892, USA; 4 The Buck Institute for Research on Aging, Novato, CA, 94945, USA; 5 Rutgers University, Dept. of Molecular Biology and Biochemistry, Piscataway, NJ, 08854, USA

## Abstract

The *Caenorhabditis* Intervention Testing Program (CITP) was founded on the principle that compounds with positive effects across a genetically diverse test-set should have an increased probability of engaging conserved biochemical pathways with mammalian translational potential. To fulfill its mandate, the CITP uses a genetic diversity panel of *Caenorhabditis *strains for assaying longevity effects of candidate compounds. The panel comprises 22 strains from three different species, collected globally, to achieve inter-population genetic diversity. The three represented species, *C. elegans*, *C. briggsae*, and *C. tropicalis*, are all sequential hermaphrodites, which simplifies experimental procedures while maximizing intra-population homogeneity. Here, we present estimates of the genetic diversity encapsulated by the constituent strains in the panel based on their most recently published and publicly available whole-genome sequences, as well as two newly generated genomic data sets. We observed average genome-wide nucleotide diversity (π) within the *C. elegans *(1.2e-3), *C. briggsae *(7.5e-3), and *C. tropicalis* strains (2.6e-3) greater than estimates for human populations, and comparable to that found in mouse populations. Our analysis supports the assumption that the CITP screening panel encompasses broad genetic diversity, suggesting that lifespan-extending chemicals with efficacy across the panel should be enriched for interventions that function on conserved processes that are shared across genetic backgrounds. While the diversity panel was established by the CITP for studying longevity interventions, the panel may prove useful for the broader research community when seeking broadly efficacious interventions for any phenotype with potential genetic background effects.

**Figure 1. Geographic and genetic diversity in the CITP strain panel:**
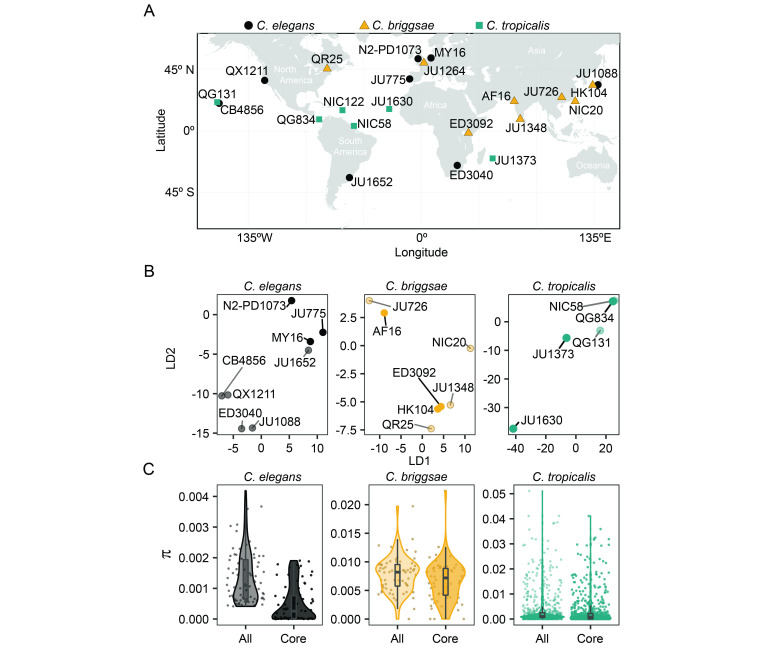
(A) The CITP diversity panel represents 22 total nematode strains from three species, *C. elegans* (black circles), *C. briggsae* (orange triangles), and *C. tropicalis* (teal squares). (B) Two-dimensional representation of diversity and relatedness of the strains, LD1 and LD2 represent two latent dimensions. Bright colors indicate the core CITP strains (*C. elegans*:N2-PD1073, JU775, MY16; *C. briggsae*: AF16, HK104, ED3092; and *C. tropicalis*:JU1373, JU1630, QG843), while all other strains in the CITP diversity panel are indicated by muted colors. (C) Nucleotide diversity (π) of the strains used in the CITP diversity panel estimated on non-overlapping 100 kb genomic windows, core strains demonstrated by bright colors, all other strains of the panel in muted colors. The grey boxes represent the mean and the standard deviations*.*

## Description

Model organisms have been fruitful tools for elucidating core biological principles. The power of model organism study, in part, is due to the ability to grow large populations with known genetic makeup. One of the most widely adopted genetic models is the hermaphroditic nematode *Caenorhabditis elegans*. The reproductive style of *C. elegans* makes it particularly easy to generate and maintain large populations of genetically identical individuals. In fact, the control over genetic variability helped make *C. elegans* the first multi-cellular organism to have its entire genome sequenced (*C. elegans* Sequencing Consortium 1998). Despite the success garnered using genetically homogeneous populations, it has become increasingly apparent that many of the phenotypes of interest for study are dependent on genetic background (see Evans *et al.* 2021 for review). Examples of these background-influenced phenotypes range from α-synuclein toxicity (Wang *et al.* 2019), to behavioral responses to temperature (Stegeman *et al.* 2013), dietary influence on lifespan and reproduction (Stastna *et al.* 2015), and pharmacological efficacy (Lucanic *et al.* 2017). The dependence on genetic background suggests that attempts to identify core biological systems and functionality could benefit from assaying across genetic diversity to identify genetic background-independent phenotypes. One way to achieve this is to use a panel of populations with intra-population homogeneity and inter-population diversity.

With the importance of genetic background effects in mind, the *Caenorhabditis* Intervention Testing Program (CITP) was designed to identify anti-aging and longevity-promoting compound interventions effective in a genetically diverse set of *Caenorhabditis* nematode populations (Lucanic *et al.* 2017). To date, 55 chemical compounds have been tested for reproducible, genetic background-independent effects on longevity (Lucanic *et al.* 2017; Banse *et al.* 2019; Coleman-Hulbert *et al.* 2019; Coleman-Hulbert *et al.* 2020; Morshead *et al.* 2020; Osman *et al.* 2021; Banse *et al.* 2021; Onken *et al.* 2021). The genetic diversity panel used by the CITP is composed of 22 strains from three hermaphroditic species, which facilitates maintenance of intra-population homogeneity. While the full panel is composed of 22 stains, a smaller core sub-panel of nine strains (three from each of three species, *C. elegans* (N2-PD1073, JU775, MY16), *C. briggsae* (AF16, HK104, ED3092), and *C. tropicalis* (JU1373, JU1630, QG843) is used in initial compound effect characterization (Banse *et al.* 2021). Here, we present estimates of the genetic diversity encapsulated by the constituent strains of the CITP panel, and core sub-panel, based on their most recently published and publicly available whole-genome sequences.

When establishing the genetic diversity panel for compound screening, the CITP sought strains that represented both broad geographic and genetic diversity. The three species represented in the panel are themselves globally distributed, but with ecological restrictions. For example, *C. elegans* is typically isolated from cooler ecological niches than *C. tropicalis* (Kiontke *et al.* 2011; Frézal and Félix 2015; Noble *et al.* 2021), while *C. briggsae* is found in niches that range from cool to warm (Frézal and Félix 2015). The wide species distributions enabled the CITP to assemble a panel of strains isolated worldwide, with representatives from most continents (Figure 1A). We next sought to determine the genetic diversity encapsulated by the panel by collecting genomic data for the strains (see Reagents) and analyzing the genomes for variation within each species (see Software). To visualize the population structure within the panel for each species we used a variational autoencoder approach, popVAE (Battey *et al.* 2021), to project genotypes of the strains on two latent dimensions (Figure 1B). We then determined the nucleotide diversity in the panel. The observed average genome-wide nucleotide diversity (π) among the *C. elegans*, *C. briggsae*, and *C. tropicalis* strains were, respectively, 1.2e-3, 7.5e-3, and 2.6e-3 (3.8e-4, 6.2e-3, and 2.4e-3 for the nine core CITP strains), which is consistent with previous estimates for those species (Graustein *et al.* 2002; Jovelin *et al.* 2009; Andersen *et al.* 2012; Crombie *et al.* 2019; Noble *et al.* 2021). Figure 1C shows nucleotide diversity estimated on 100 kb windows along the genomes for both the 20 strains in the full panel with available sequencing data, and for the nine strains in the core sub-panel. The estimated level of genetic diversity within these *Caenorhabditis* species is higher than that within human populations (Yu *et al.* 2004; Perry *et al.* 2013; Prado-Martinez *et al.* 2013; Arbiza *et al.* 2014; 1000 Genomes Project Consortium *et al.* 2015) and comparable to that found in mouse populations (Halligan *et al.* 2010; Booker and Keightley 2018). Our analysis supports the assumption that the CITP screening panel encompasses broad genetic diversity, suggesting that lifespan-extending chemicals with efficacy across the panel should be enriched for interventions that function on conserved processes that are shared across genetic backgrounds. While the diversity panel was established by the CITP for studying longevity interventions, the panel may prove useful for the broader research community when seeking broadly efficacious interventions for any phenotype with potential genetic background effects.

## Methods

To generate estimates of the genetic variability among the strains in the CITP diversity panel we collected publicly available genomic data for 18 of the 22 CITP strains (see Reagents below). Because comparable Illumina-based sequencing was unavailable for N2-PD1073 and ED3092, two strains present in the core-subpanel of nine strains, we generated whole genome data for these two strains using standard protocols. In brief, we used proteinase K to digest whole worms and isolated total genomic DNA using the Genomic DNA Clean & Concentrator kit (Zymo Research). Genomic libraries were then generated using the Nextera XT DNA Library Preparation Kit (Illumina, Inc.). Sequencing was carried out on the Illumina NovaSeq 6000 platform by the Genomics and Cell Characterization Core Facility (GC3F) at the University of Oregon. We then used the two new genomic datasets, along with the 18 available in the NCBI database, to calculate genetic diversity estimates using our software pipeline (described below).

## Reagents

While most of the strains in the CITP diversity panel can be obtained from the *Caenorhabditis* Genetics Center (CGC), the *C. elegans* wild-isolates should be ordered from the *Caenorhabditis elegans* Natural Diversity Resource (CeNDR) (Cook *et al.* 2017). Strains unavailable from those sources (e.g., N2-PD1073) can be obtained directly from the CITP upon request. Relevant to the unavailability of PD1073 at the CGC, PD1073 is a close relative of PD1074 which is distributed by the CGC as a wild type reference strain. PD1073, PD1074, and PD1075 are three subclones of the N2-derivative strain VC2010 that were generated in the process of assembling a new N2 reference (“VC2010-1.0”) genome (Yoshimura *et al.* 2019). All strains in the genetic diversity panel and the associated SRA read accession IDs used in this study are listed below:

*C. elegans*: strain CB4856 (with SRA read accession numbers SRR9322768, SRR9322769, SRR9322775, SRR9322863, SRR9322864), ED3040 (SRR9322720, SRR9322724, SRR9322725, SRR9322726, SRR9322818), JU1088 (SRR9322295, SRR9322300, SRR9322301), JU1652 (SRR9322577, SRR9322578, SRR9322579), JU775 (SRR9322349, SRR9322352, SRR9322355, SRR9322364), MY16 (SRR9322907, SRR9322327, SRR9322913, SRR9322161), QX1211 (SRR9324168, SRR9324217, SRR9323954, SRR9323955);

*C. briggsae*: AF16 (SRR2002620), HK104 (ERR3063412, ERR3063411, SRR8333803), JU1348 (SRR1793004), JU726 (SRR1792964), NIC20 (SRR1793012), QR25 (SRR1793006);

*C. tropicalis*: JU1373 (SRR12623131, SRR241785), JU1630 (SRR12623130), NIC58 (SRR12623045, SRR12623063), QG131 (SRR12623047), QG834 (SRR12623046).

No genomic data is publicly available for *C. briggsae* strain JU1264 and *C. tropicalis* NIC122.

Raw reads for *C. elegans* N2-PD1073 and *C. briggsae* ED3092 strains generated in this study were deposited to the SRA database (https://www.ncbi.nlm.nih.gov/sra) under the project accession ID PRJNA773598.

The core CITP strains are *C. elegans* N2-PD1073, MY16, and JU775; *C. briggsae* AF16, ED3092, and HK104; *C. tropicalis* JU1373, JU1630, and QG834.

**Software**

We estimated genetic diversity of the CITP strains using whole-genomic data for 20 out of 22 CITP strains from the panel. Reads from the SRA database were downloaded with SRA-toolkit (the SRA Toolkit Development Team). We evaluated the read quality with FastQC v.0.11.5 (Andrews 2010) and MultiQC v.1.3 (Ewels *et al.* 2016), and filtered and trimmed reads with Skewer v.0.2.2 (Jiang *et al.* 2014). We mapped filtered reads to the *C. elegans*, *C. briggsae*,and *C. tropicalis* genomes obtained from the WormBase database (https://wormbase.org//) version WS280 (with accession ID PRJNA13758, PRJNA10731, PRJNA53597, respectively) by BWA-MEM v.0.7.17 (Li 2013) and SAMtools v.1.5 (Li *et al.* 2009), and deduplicated with the Picard tools v.2.17.6 (Broad Institute). We called and filtered variants using GATK v.3.7 (McKenna *et al.* 2010) software and BEDtools v.2.25 (Quinlan and Hall 2010), and estimated the nucleotide diversity using VCFtools v.0.1.15 (Danecek *et al.* 2011), masking repetitive regions, indels, complex variants, and regions with too low or high coverage. We projected genotypes of the CITP strains on two latent dimensions using popVAE (Battey *et al*. 2021), and visualized them in R v.3.5 (R Core team 2018) using packages ggplot2 (Wickham 2016) and ggrepel.

All the tools used for the analysis are publicly available and free, the scripts used to get the results and figures are in the GitHub repository (https://github.com/phillips-lab/CITP_diversity/) and are available under the MIT license. The global map was generated in MATLAB R2021b (MathWorks, Inc.) using the MATLAB Mapping Toolbox. The location coordinates used for the strains are included in the GitHub repository. Figure readability was improved by moving panel relative positions, updating color coding, and improving text aesthetics using Adobe Illustrator 2022 in a manner consistent with image integrity standards.
